# ﻿*Graphidessajinfoensis*, a new species of longhorned beetle (Coleoptera, Cerambycidae, Lamiinae, Desmiphorini) from China

**DOI:** 10.3897/zookeys.1186.112377

**Published:** 2023-12-07

**Authors:** Chuan Liu, Zhentao Cheng, Yongchuan Yang, Xiaolei Huang

**Affiliations:** 1 State Key Laboratory of Ecological Pest Control for Fujian and Taiwan Crops, College of Plant Protection, Fujian Agriculture and Forestry University, Fuzhou 350002, China Fujian Agriculture and Forestry University Fuzhou China; 2 Key Laboratory of the Three Gorges Reservoir Region’s Eco-Environment, Ministry of Education, Chongqing University, Chongqing 400045, China Chongqing University Chongqing China

**Keywords:** Chongqing, COI gene, genitalia, key, taxonomy

## Abstract

*Graphidessajinfoensis***sp. nov.** is described from Chongqing and Guizhou in Southwest China. The diagnostic morphological characters of the new species are described and illustrated in color plates. The distribution of all species of the genus *Graphidessa* Bates, 1884 is mapped and the key to all species of this genus is updated. The *COI* gene sequence of the new species is also provided.

## ﻿Introduction

*Graphidessa* Bates, 1884 is a small genus belonging to the subfamily Lamiinae Latreille, 1825. This genus is characterized by legs covered with abundant yellowish-white pubescence and long black setae, and each elytron has longitudinal haired stripes and a strongly tuberculate base. *Graphidessa* currently contains three described species and one subspecies (Bates 1884; Hayashi 1974; Komiya and Kusama 1974; Fujita 1980), and all of them were recorded only in East Asia. *Graphidessaobliquefasciata* Komiya & Kusama, 1974 and *Graphidessavariegata* Hayashi, 1974 were recorded in Taiwan Island, China (Hayashi 1974; Komiya and Kusama 1974); *Graphidessavenatavenata* Bates, 1884 and *Graphidessavenatatakakuwai* Fujita, 1980 were recorded in Japan (Bates 1884; Fujita 1980).

## ﻿Material and methods

One male specimen and one female specimen used in this study were acquired from the project: Biodiversity along Elevational gradients: Shifts and Transitions (BEST). Malaise traps were used in the project to collect insects along the elevation gradient of the Jinfoshan National Nature Reserve, Chongqing, China. The other two female specimens used were collected from Guizhou, China. The collected specimens were carefully preserved in 95% alcohol and stored at -20 °C. The whole genomic DNA was extracted from the abdomen of the male specimen using a Mirco Cell/Tissue DNA Kit (Biomarker Technologies), following the revised operation steps of the manufacturer’s manual: each sample was pierced with a hole on the abdomen with a fine needle, and incubated in lysis buffer for at least 12 hours on a constant temperature shaker to fully split the DNA; the centrifuge column was kept at room temperature for 3 mins before adding Elution Buffer.

The barcoding region of *COI* (mitochondrial cytochrome c oxidase subunit I) was amplified using the following primers: LCO1490 (5’-GGTCAACAAATCATAAAGATATTG-3’) and HCO2198 (5’-TAAACTTCAGGGTGACCAAAAAAT-3’) (Folmer et al. 1994). The polymerase chain reaction (PCR) amplifications were performed using a 25 μL reaction volume with the mixture of 1 μL template DNA, 0.5 μL forward and reverse primer (10 μM), 0.25 μL Taq DNA polymerase (5 U/μL), 18.25 μL double distilled H_2_O, 2.5 μL 10× buffer and 2 μL dNTP. The *COI* fragment amplification was carried out with the following conditions: 5 min of initial denaturation at 95 °C, 35 cycles of 20s at 94 °C, 30s at 54 °C (the annealing temperature) and 72 °C at 2 min, and 10 min of final extension at 72 °C. The products of PCR were run in 1% agarose gels and bidirectionally sequenced at Sangon Biotech (Shanghai, China). Geneious R11 (Auckland, New Zealand) was used to check the quality of the chromatograms, and export them as FASTA format. The COI sequence was submitted to GenBank under the accession number: OR366841.

The description of the new species was made using a Leica M165C stereo microscope, aided by Leica LED 5000 HDI dome light source. Images were captured using a Leica MC170 HD digital camera attached to the microscope, and subsequent measurements of the specimens, including body length and antennae, were conducted using Leica MC170 software. Serial images were aligned and merged using Zerene Stacker (www.zerenesystems.com) software, while Adobe Photoshop 2021 (www.adobe.com) was employed to refine and enhance the visual clarity of the images. The distributions of *Graphidessa* species were obtained and annotated on the map using the online tool SimpleMapper.

The holotype and one of the paratypes are deposited in the Insect Systematics and Diversity Lab, Fujian Agriculture and Forestry University (FAFU), Fuzhou, China, and the two remaining paratypes are deposited in the School of Biological Science and Technology, Liupanshui Normal University, Liupanshui, Guizhou, China (LPSNU).

## ﻿Results

**Figure 1.**
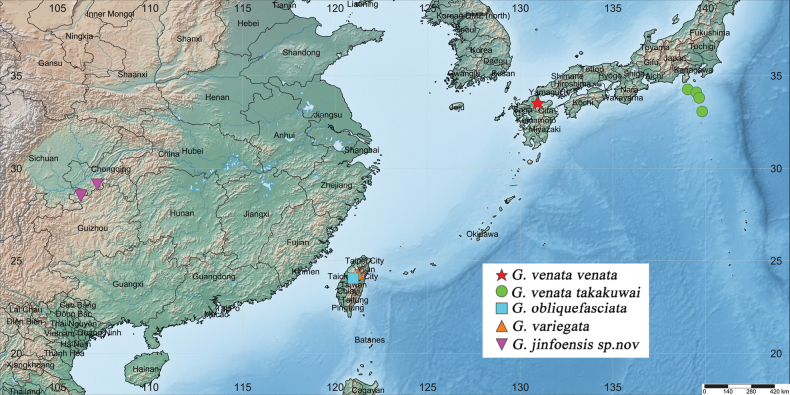
Distribution of *Graphidessa* species.

*Graphidessajinfoensis* sp. nov. is described based on the specimens collected in the Jinfoshan National Nature Reserve of Chongqing and Dongfeng Lake National Wetland Park of Guizhou in Southwest China, which constitute the first specimen records of the genus *Graphidessa* from mainland China. The genus *Graphidessa* now consists of four species (including one species with two subspecies), three of which are distributed in China (Fig. 1).

### ﻿ Graphidessa jinfoensis sp. nov.

Taxon classificationAnimaliaColeopteraCerambycidae

89ACAA0E-0B65-5A54-9421-6027BCB61164

https://zoobank.org/C28A8611-9D3A-4E01-8750-54F638FC47AD

**Figure 2.**
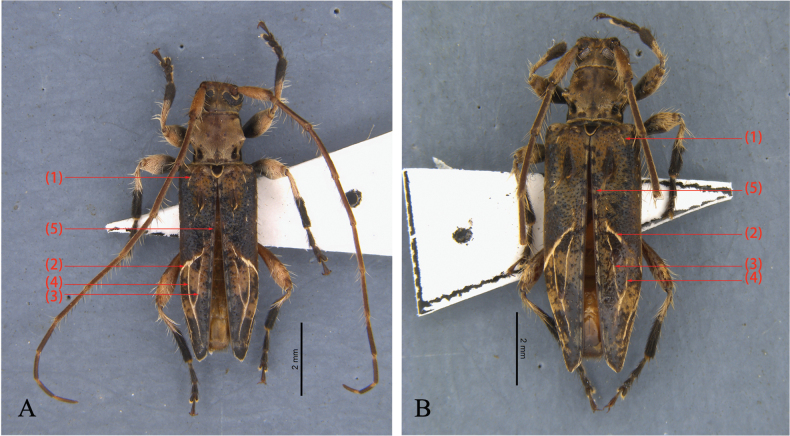
*Graphidessajinfoensis* sp. nov. habitus, dorsal view **A** holotype, male **B** paratype, female.

**Figure 3.**
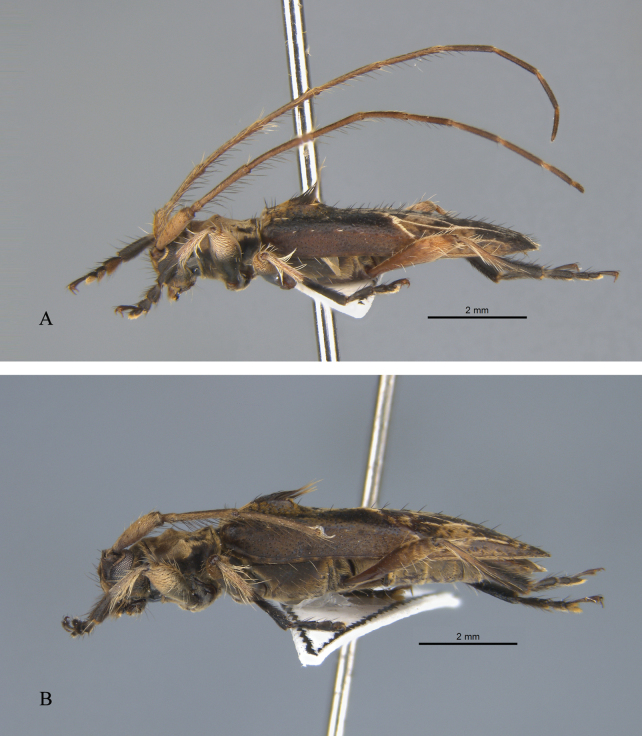
*Graphidessajinfoensis* sp. nov., habitus, lateral view **A** male (holotype) **B** female (paratype).

**Figure 4.**
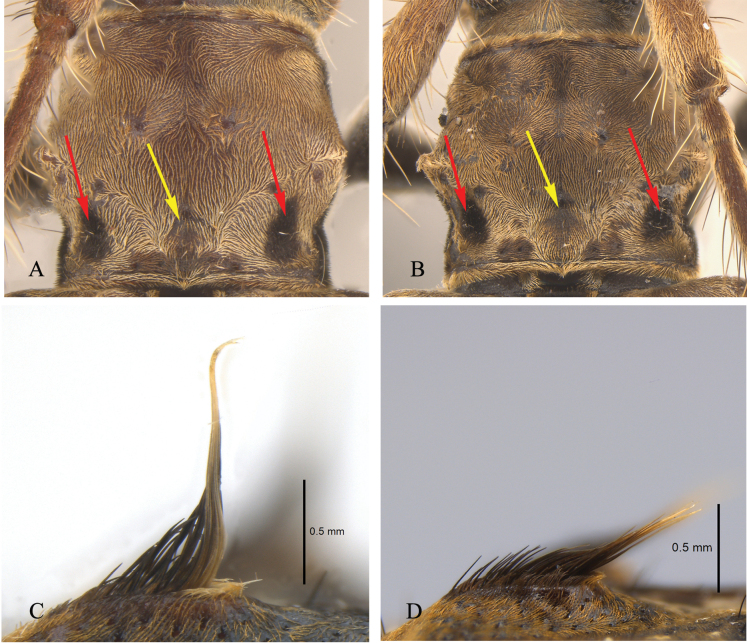
*Graphidessajinfoensis* sp. nov. **A, B** details of pronotum **C, D** details of the bump near base of the elytra **A, C** male (holotype) **B, D** female (paratype), not to scale (**A, B)**.

**Figure 5.**
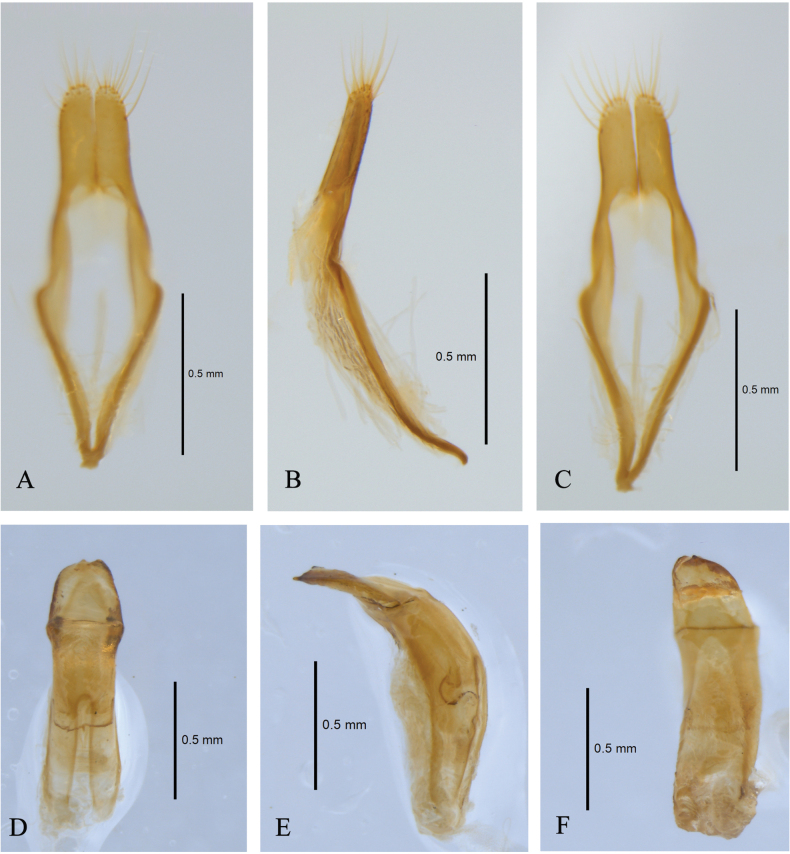
*Graphidessajinfoensis* sp. nov. holotype, male genitalia **A**–**C** tegmen **D**–**F** median lobe (**A, D** dorsal view **B, E** lateral view **C, F** ventral view).

**Figure 6.**
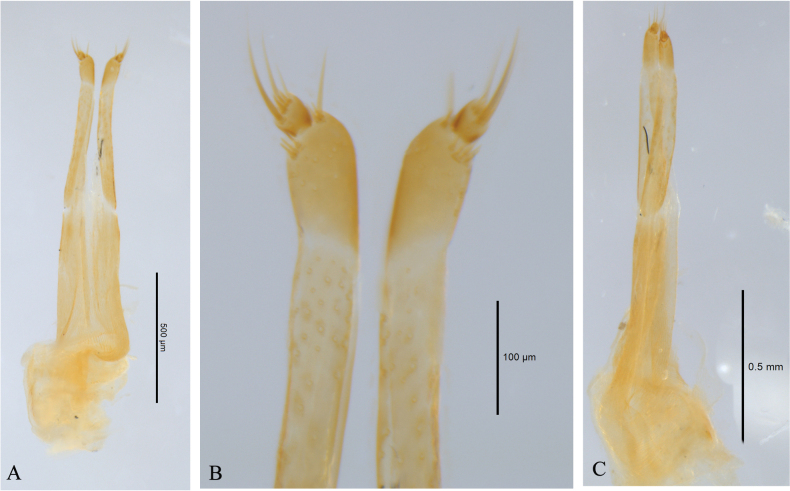
*Graphidessajinfoensis* sp. nov., paratype, female ovipositor **A** dorsal view **B** showing ovipositor dorsal view details **C** lateral view.

**Figure 7.**
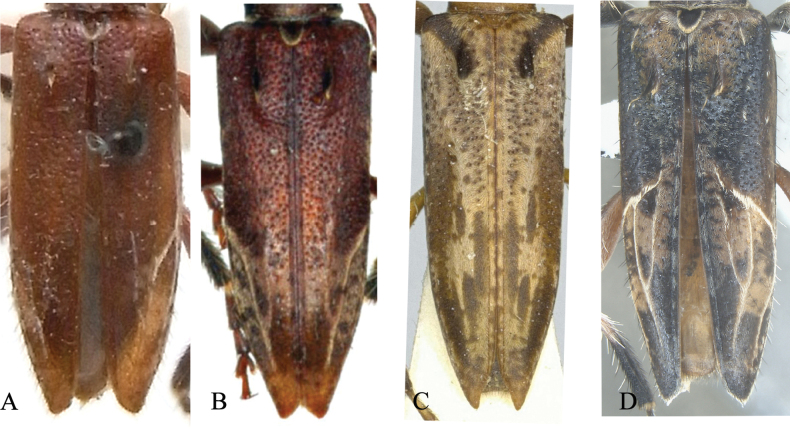
Dorsal view of elytra **A***Graphidessavenatavenata* Bates, 1884 ♂ holotype **B***Graphidessavenatatakakuwai* Fujita, 1980 ♂ holotype **C***Graphidessavariegata* Hayashi, 1974 ♂ holotype **D***Graphidessajinfoensis* sp. nov. ♂ holotype.

Figs 1 , 2 , 3 , 4 , 5 , 6 , 7D

#### Type materials examined.

Specimen code: CQ114714. ***Holotype***, ♂, glued on paper point, with genitalia in a separate centrifugal tube. Original label: “中国重庆金佛山国家级自然保护区,2022年10月30日,马氏网,周礼华采” [Jinfoshan National Nature Reserve, Chongqing, China, 2022.X.18, malaise trap, Lihua Zhou leg. (FAFU)], HOLOTYPE / Graphidessa / Jinfoensis / Xiaolei Huang Geanbank accession number: OR366841 [handwritten red label]. ***Paratype***: 1♀, Original label: “中国重庆金佛山国家级自然保护区,2022年5月15日,马氏网,周礼华采” [S14, Jinfoshan National Nature Reserve, Chongqing, China, 2022.V.15, malaise trap, Lihua Zhou leg. (FAFU)]; 2♀♀, Dongfeng Lake National Wetland Park, Xishui County, Zunyi City, Guizhou Province, China, 23.X.2022, leg. Xiudong Huang (LPSNU).

#### Diagnosis.

The new species can be distinguished from its closest congeners by the dark brown body, densely covered with short chestnut-like pubescence, and the unique elytral pattern. Head and most of the pronotum are densely covered with short brown hairs, the ventral surface of the prothorax is black, the dorsum of the prothorax is brown with some black patches. Elytra dark brown and covered with short brown pubescence, and black, brown and grayish setae; the white pubescence forming five stripes on each elytron. The bump near elytral base is covered with many long setae, the black setae shorter than the brown setae.

#### Description.

**Male** (Figs 2A, 3A), holotype. ***Body length***: 6.2 mm (*N* = 1). ***Head***: brown, covered with short brown hairs. Forehead covered with long pale brown setae near mouthpart. Scape densely covered with short brown hairs and a few dark brown and yellowish setae; antennomeres II–VII covered with densely short brown hairs and sparsely long black setae beneath; antennomeres VIII–XI covered with short, dense brown hairs. Each antennomere with a brown recumbent seta apically. Length ratio of antennomeres from base to tip: 4.7: 1: 5.4: 6.7: 5.9: 5.5: 5.1: 4.1: 3.4: 3.5: 3.3 (Figs 2A, 3A). ***Thorax***: ventral surface of thorax with brown pubescence, interspersed with a few long, erect brownish setae. Two small patches of dark brown short pubescence near the posterior margin of the anterothoracic backplane (two small patches on each side, they are marked with red arrows in Fig. 4A) three petal-like patches of light brown short pubescence precede peltate (three petal-like patches are between the two patches. Three petal-like patches are marked with yellow arrows in Fig. 4A). Prosternum black. (Figs 2A, 4A).

***Legs***: all legs with abundant yellowish-white pubescence and black setae. The basal half of pro- and mesofemora mostly black, covered with short, dense brown hairs and interspersed with a few long erect brownish setae; the apical half of pro- and mesofemora covered with densely short yellowish-white hairs dorsally and interspersed with a few long erect yellowish-white setae. Basal half of pro- and mesotibiae with yellowish-white pubescence, obscuring integument, and some setae. Apical half of tibiae with black pubescence obscuring integument and a few long, erect black setae interspersed. Metatarsomere I 0.97 times as long as II–III together (Figs 2A, 3A). ***Elytra***: sparsely covered with erect setae, generally black, with white, golden, and brownish pubescence interspersion. Elytra dark areas more than half, light color areas sparsely covered with golden pubescence, dark areas with fewer golden pubescence. Strongly tuberculate at base covered with long and dense setae, posterior setae longer than anterior ones (bump near elytral base covered with long and dense black setae anteriorly, with dense yellowish-brown setae posteriorly; yellowish-brown setae much longer than black setae, up to 13 mm); disc about 2.71 times as long as humeral width.

Each elytron covered with five longitudinal haired stripes, the first stripe yellowish white, starting from the middle of the basal of elytron, and about one-seventh of elytron length; the second stripe yellowish white, starting at the middle of elytron near suture, extending to the two-thirds of elytron length near outer margin, and ends near outer apical angle; the third yellowish white, starting at the base of the second stripe, extending straight toward outer apical angle, and stops at half of the elytra width; the fourth stripe yellowish white, starting at the intersection of the centerline of elytron and the second stripe, and joins to the third stripe at the middle; a series of short greyish white haired stripes form the fifth stripe, starting from posterior of scutellum and extending along the suture to inner apical angle. Outer margin with some off-white long sub-erect grayish white setae. ***Abdomen***: abdominal ventrites covered with long and sparse brown hairs. ***Male genitalia*** (Fig. 5): tegmen (Fig. 5A–C), lateral lobes near parallel, then gradually narrowing towards apices, rounded and sparsely covered with long brown setae. Median lobe (Fig. 5D–F) moderately curved in profile, median struts about 0.5 times as long as median lobe, ventral plate truncated at apex, dorsal plate narrower than ventral plate, slightly concave at apical middle. ***Female genitalia*** (Fig. 6A–C): with ovipositor elongate, narrow, apically with short styli.

**Female** (Figs 2B, 3B), paratypes. ***Body length***: 7.5–9 mm (*N* = 3) Similar to male, but elytra about 2.64 times as long as humeral width; bump near elytral base covered with shorter yellowish-brown setae up to 8 mm.

#### Etymology.

The scientific name is derived from the Jinfoshan National Nature Reserve, where the holotype was collected.

#### Distribution.

China: Chongqing, Guizhou (Fig. 1).

#### Remarks.

Left antennomeres V–XI were separated from antennomere IV for one paratype from Guizhou. Right antennomeres IV–XI were missing, the black and yellowish-brown setae on the bump near the elytral base were worn off, right hind leg was separated from body in another paratype from Guizhou.

## ﻿Discussion

Information on *Graphidessaobliquefasciata* is notably scarce in online databases like GBIF, Catalogue of Life, Taiwan Encyclopedia of Life and Titan (Tavakilian and Chevillotte 2023). The holotype of this species also cannot be traced in museums worldwide. Moreover, despite multiple attempts, no images of the type specimen of *G.obliquefasciata* could be obtained. Therefore, in this paper, the description of *G.obliquefasciata* was solely derived from the original literature (Komiya and Kusama 1974). In order to obtain more details on the morphology, ecology and bionomy of this species, more extensive sampling should be conducted in the future.

The picture of a male of *G.variegata* in the Atlas of Cerambycidae of Taiwan (Chou, 2008) looks similar to the male of *G.jinfoensis* sp. nov. However, after comparison, we found that the pattern of the elytral stripe (3) and the color of the hind femora of the male specimen shown in Chou’s Atlas are very different from *G.jinfoensis* sp. nov. The elytral stripe (3) in that specimen is disconnected, and the color of the hind femora are dark brown, while the overall color of the body is light brown. On the contrary, the elytral stripe (3) in *G.jinfoensis* sp. nov. is continuous, and the hind femora are light brown, with many yellowish long setae and soft hairs. And the body color of *G.jinfoensis* sp. nov. is dark brown (Fig. 2). Regarding the morphological comparison with the holotypes of the remaining described *Graphidessa* species, there is a reasonable suspicion that the male sample named as *G.variegata* in *The Atlas of Cerambycidae of Taiwan* represents a yet undescribed taxon.

### ﻿Key to species of *Graphidessa* Bates, 1884

**Table:** 

1	Each elytron with less than five stripes	**2**
–	Each elytron with five stripes	**3**
2	Each elytron covered at the base with an oblique short black-haired band basally	***G.variegata***
–	Each elytron not covered at the base with an oblique short black-haired band basally	**4**
3	Each elytron with five yellowish stripes, body dark brown	***G.jinfoensis* sp. nov**
–	Each elytron with five blackish stripes, body light reddish brown	***G.obliquefasciata***
4	Elytra with relatively sparse yellow-white short hairs	***G.venatavenata***
–	Elytra with relatively dense yellow-white short hairs	***G.venatatakakuwai***

## Supplementary Material

XML Treatment for Graphidessa jinfoensis

## ﻿Additional information

### Author ORCIDs

Chuan Liu https://orcid.org/0009-0004-3849-6280

Zhentao Cheng https://orcid.org/0000-0003-3452-4075

Yongchuan Yang https://orcid.org/0000-0001-7627-7776

Xiaolei Huang https://orcid.org/0000-0002-6839-9922

### Data availability

All of the data that support the findings of this study are available in the main text
